# Sustaining Vaccine Confidence in the 21st Century

**DOI:** 10.3390/vaccines1030204

**Published:** 2013-06-24

**Authors:** Karin Hardt, Ruprecht Schmidt-Ott, Steffen Glismann, Richard A. Adegbola, François P. Meurice

**Affiliations:** 1 GlaxoSmithKline Vaccines, Global Scientific Affairs & Medical Education, 20 Avenue Fleming, 1300 Wavre, Belgium; E-Mails: richard.a.adegbola@gsk.com (R.A.A.); francois.meurice@gsk.com (F.P.M.); 2 GlaxoSmithKline Vaccines, Global Scientific Affairs & Medical Education, Prinzregentenplatz 9, 81675 Munich, Germany; E-Mail: ruprecht.r.schmidt-ott@gsk.com; 3 GlaxoSmithKline Vaccines, Global Scientific Affairs & Medical Education, 68 Nykaer, DK-2605 Broendby, Denmark; E-Mail: steffen.x.glismann@gsk.com

**Keywords:** vaccine safety, vaccine confidence, vaccine hesitancy, public health, immunization, coverage, pharmaceutical industry

## Abstract

Vaccination provides many health and economic benefits to individuals and society, and public support for immunization programs is generally high. However, the benefits of vaccines are often not fully valued when public discussions on vaccine safety, quality or efficacy arise, and the spread of misinformation via the internet and other media has the potential to undermine immunization programs. Factors associated with improved public confidence in vaccines include evidence-based decision-making procedures and recommendations, controlled processes for licensing and monitoring vaccine safety and effectiveness and disease surveillance. Community engagement with appropriate communication approaches for each audience is a key factor in building trust in vaccines. Vaccine safety/quality issues should be handled rapidly and transparently by informing and involving those most affected and those concerned with public health in effective ways. Openness and transparency in the exchange of information between industry and other stakeholders is also important. To maximize the safety of vaccines, and thus sustain trust in vaccines, partnerships are needed between public health sector stakeholders. Vaccine confidence can be improved through collaborations that ensure high vaccine uptake rates and that inform the public and other stakeholders of the benefits of vaccines and how vaccine safety is constantly assessed, assured and communicated.

## 1. Introduction

Vaccines have made enormous contributions to public health allowing, for example, for the global eradication of small pox and elimination of poliomyelitis from most countries [1]. Levels of support for childhood vaccinations have improved, as demonstrated by worldwide coverage in 2010 with the third dose of diphtheria-tetanus-pertussis (DTP) vaccine, Bacille Calmette–Guérin (BCG) vaccine, the third dose of poliovirus vaccine and the first dose of measles-containing vaccine, which was estimated to be 85% or higher among young children, representing at least 109.4 million immunized children on an annual basis [2]. Table 1 summarizes the impact of vaccines in the USA [3]; worldwide, with childhood vaccination, approximately three million lives are saved annually [1] and millions of disease episodes and disabilities are avoided each year [4]. Established immunization programs have provided many economic benefits for individuals, their families and society [5,6]. 

**Table 1:** Impact of vaccines in the USA in terms of numbers of reported cases and deaths associated with disease before and after the introduction of vaccination (reprinted and adapted from Bonanni and Santos 2011 [7] and Roush and Murphy 2007 [3].

Disease	Pre-vaccination (estimated annual average)		Post-vaccination (year)	
	Cases	Deaths	Cases	Deaths
Diphtheria	21,053	1,822	0 (2006)	0 (2004)
Measles	530,217	440	55 (2006)	0 (2004)
Mumps	162,344	39	6,584 (2006)	0 (2004)
Pertussis	200,752	4,034	15,632 (2006)	27 (2004)
Poliomyelitis, acute	19,794	1,393	0 (2006)	0 (2004)
Poliomyelitis, paralytic	16,316	1,879	0 (2006)	0 (2004)
Rubella	47,745	17	11 (2006)	0 (2004)
Congenital rubella syndrome	152	Not available	1 (2006)	0 (2004)
Smallpox	29,005	337	0 (2006)	0 (2004)
Tetanus	580	472	41 (2006)	4 (2004)
Hepatitis A	117,333	137	3,579 (2006)	18 (2006)
Acute hepatitis B	66,232	237	4,713 (2006)	47 (2006)
Invasive Hib	20,000	1,000	208 (2006)	<5 (2005)
IPD	63,067	6,500	5,169 (2006)	4,850 (2005)
Varicella	4,085,120	105	48,445 (2006)	19 (2004)

Hib = *Haemophilus influenzae* type b; IPD = invasive pneumococcal disease.

Despite these benefits, many children and adults are not vaccinated [4,8]. Annually, 19.3 million children from the world’s poorest settings do not receive vaccines, such as DTP [9]. This is recognized by a collaboration of supranational organizations that have named the period 2011 to 2020 the ‘Decade of Vaccines’, with the mission of extending, by 2020 and beyond, the full benefit of immunization to all people, regardless of where they are born, who they are or where they live [10,11]. Significantly scaling up the delivery of vaccines through the introduction of new vaccines and encouraging countries to reach 90% coverage might save the lives of 8.7 million children aged under five years during this decade [12]. 

Suboptimal vaccination rates are observed not only in developing countries, but also in industrialized regions [4,13,14,15]. This has consequences not only for direct protection of the vaccinated individual, but also for population herd protection, whereby a majority of immunized subjects prevents circulation of infectious agents in the remaining unvaccinated susceptible population [16]. Low vaccination rates may result from a lack of infrastructure or resources, but also from low vaccine confidence. Reasons for the latter include concerns from parents or guardians and healthcare providers about vaccines, most frequently vaccine safety. Vaccines are usually given to large numbers of healthy people in order to prevent disease, which is different from the use of most medicines, which are generally used to treat or control diseases [17]. Since vaccine recipients are healthy and often young children, there is a lower level of tolerance for the risk of adverse events than with other medicines. Vaccine-related adverse events are mostly time-limited and mild [17], most commonly local reactions at the injection site (pain, swelling or reddening), fever and irritability [18]. Rare reactions to vaccination, such as convulsions, thrombocytopenia, episodes of hypotonia and hyporeactivity and inconsolable persistent crying, are usually characterized by spontaneous remission with no sequelae, but can also have a significant impact on health. Anaphylaxis is another rare severe vaccine-related event that can be fatal unless treated in a timely manner [18]. Fear of such reactions can deter people from having themselves or their children vaccinated. Furthermore, as most of the diseases against which vaccines protect are no longer visible, the risks associated with the diseases are often forgotten and the need for immunization programs to control the diseases may be underappreciated [13]. Parents, when considering vaccination, may therefore worry more about possible adverse events than they do about the risks associated with exposure to disease [19]. 

Other reasons for questioning vaccines are driven by a variety of social and behavioral factors related to complex cultural issues and belief systems [20,21,22,23,24,25,26]. This may include an influence of religious or ethnic affiliation on the perceptions of disease, vaccines and authority or local practices for medical decision-making and vaccine delivery [20,22]. Other factors are related to individuals’ need for control in making thoughtful vaccination decisions for themselves and their dependents [23,24]. Mistrust in the information provided by the pharmaceutical industry and a lack of trust in the scientific research community or in government [13,14,27] may also lead to vaccine refusals. This has fostered misconceptions about vaccination, such as the belief that diseases had already begun to disappear before vaccines were introduced, because of better hygiene and sanitation or that giving a child multiple vaccines for different diseases at the same time increases the risk of harmful side effects and can overload the immune system [28]. The Internet and social media have allowed such concerns to spread rapidly and indiscriminately [29,30], and websites opposing vaccination are now prevalent, publicizing the beliefs of people with negative attitudes to vaccines to a global audience [31,32]. 

When a decrease in confidence in vaccines results in reduced coverage, the risk of disease outbreaks rises. For example, suboptimal immunization levels with measles vaccines have led to the re-emergence of measles in Europe [33,34], while a boycott of the polio vaccination campaign in Nigeria in 2003 due to public mistrust of mass immunization programs led to fresh outbreaks of polio in the region and to re-introduction of the virus into previously polio-free countries [35,36].

It is important to acknowledge and act upon vaccine-related concerns as part of the strategy to achieve high vaccine uptake rates [37]. This review examines how confidence in vaccines is attained by building on trust and by having effective vaccine safety evaluation and monitoring systems that support immunization programs. The partnerships and collaborations that are needed for sustained vaccine confidence in the 21st century are also explored. 

## 2. Factors Associated with Improved Vaccine Confidence

In a USA survey carried out in 2010, of 376 parents of children aged six years or less, 26% believed that ingredients in vaccines are unsafe and 17% felt that vaccines are not tested enough for safety; only 23% had no concerns about childhood vaccines [38]. This suggests that many people are unaware of the stringent regulatory quality and safety processes involved not only during the vaccine research, development and manufacturing phases, but also post-licensure in order to monitor and respond to safety signals that may appear during vaccine use in large populations. 

**Figure 1:**
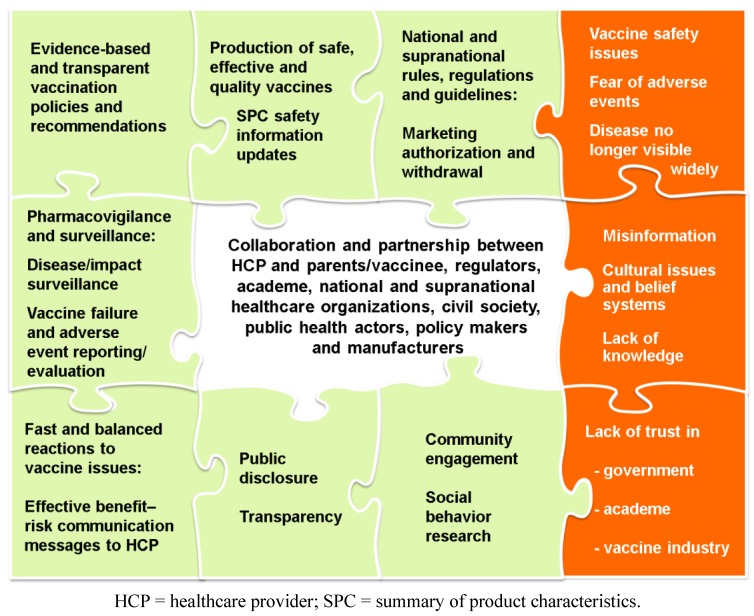
Factors that promote (outer green boxes) or undermine (outer orange boxes) vaccine confidence and collaborations (central box) associated with improved public confidence in vaccines.

However, knowledge of these safeguards is not sufficient to maintain the long-term success of immunization programs. The development of effective benefit-risk communication messages to build public trust is not straightforward, requiring input from many vaccination stakeholders. In this review, we describe the systems designed to guide and regulate vaccine development and to monitor safety and efficacy. We explore the factors involved in informing the public and others of the benefits and risks of vaccines to sustain trust in immunization programs, as well as the collaboration of different public health sector partners needed to fulfil the various roles outlined in Figure 1.

### 2.1. Vaccine Recommendations and Health Policies

Transparency in the decision-making processes for vaccine policy matters is crucial to counter the conspiracy allegations made on anti-vaccine websites regarding issues, such as government or institutional decisions about vaccine approval for licensure, public funding and safety assessments after licensing [24]. 

Each country has health policies and develops vaccine recommendations that drive national vaccination programs [39,40]. The development of national and supranational public health policies involves different levels of governments or institutions and numerous stakeholders with diverse needs and interests. Political commitment is also critical to support functional policy-making and regulatory bodies at a national level. Many countries have installed a national immunization technical advisory group, a body of national experts who advise on all technical and scientific topics related to vaccines and immunization [41] and who may elaborate recommendations on which vaccinations are appropriate in which schedule and for which population in order to help protect those at risk. In the USA, the Centers for Disease Control and Prevention (CDC) sets immunization schedules based on recommendations from the Advisory Committee on Immunization Practices (ACIP) [42,43].

Providing evidence-based vaccine recommendations and health policies that meet the needs of parents, healthcare providers and society and ensure that those working in primary care are provided with the support required to implement vaccination programs effectively should be part of immunization implementation programs.

### 2.2. Vaccine Development and Manufacturing

Vaccines are biological preparations made in, composed of and/or tested through living systems, with a mode of action that is via the immune system. They are generally administered to large numbers of healthy people to prevent disease and need thorough assessments to ensure that benefits outweigh the risks when used in the target populations [44]. As no medical intervention, including vaccines, is completely safe and without risk, this necessitates ongoing surveillance to identify safety concerns. Then, serious adverse events and associated risks can be compared to the benefits of vaccination in a benefit-risk analysis. Consequently, the processes involved in vaccine development are often complex, specific and stringent. 

**Figure 2:**
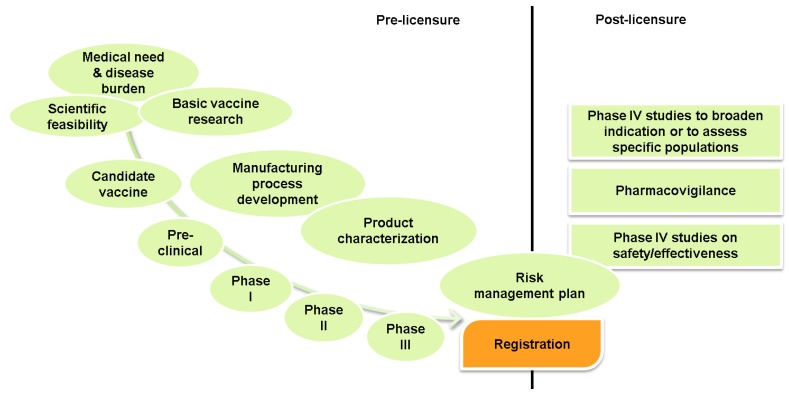
Pre- and post-licensure vaccine development activities.

Selection of a candidate vaccine is usually based on public health need (disease burden), scientific feasibility, suitable technologies and manufacturability. The development process (Figure 2) aims to deliver an efficacious vaccine with a strong and long-lasting immune response and minimal adverse effects. Once a candidate vaccine is selected, preclinical studies (*in vitro* and in animals) are conducted to provide important safety data and evaluate vaccine quality and potency [45]. This is followed by, typically, three phases in clinical development, all of which include vaccine safety assessment in their study protocols. 

Post-licensure activities are designed to monitor the impact of the vaccine in terms of immunization coverage and effectiveness in protecting against disease and safety surveillance. Manufacturers are usually required to submit risk management plans to the licensing authority that detail a set of pharmacovigilance activities and interventions to identify, characterize, prevent or minimize risks related to the vaccine they intend to market [46]. Phase IV trials are often conducted after the vaccine has been licensed to broaden the vaccine indication (e.g., altered schedule) or assess the vaccine in specific populations, such as immunosuppressed persons, low birth weight infants, chronic disease populations or pregnant women [47,48,49]. In addition, most countries have ongoing vaccine safety assessment through passive or active surveillance systems.

The vaccine manufacturing processes that ensure the end product is safe for use are complex, as they require, for instance, sterile manufacturing conditions and involve challenges that differ from those associated with non-biological drug manufacturing [50]. Each type of vaccine (e.g., live attenuated, inactivated or killed whole viruses or bacteria or subunit antigens that are naturally derived or generated using recombinant DNA technology) presents separate specificities in terms of manufacturing, analytic characterization and safety evaluation [47,51], and vaccine development and manufacturing must adhere to stringent rules and regulations, as described below.

#### 2.2.1. Rules and Regulations—Vaccine Registration and Evaluation

Currently, extensive scientific and regulatory processes are in place to ensure product quality, safety and efficacy before vaccines are licensed and made available to the public [47]. These processes are mandated by guidelines and rules from organizations, including the World Health Organization (WHO), the Food and Drug Administration (FDA) in the USA, the European Medicines Agency (EMA) and the International Conference on Harmonization of Technical Requirements for Registration of Pharmaceuticals for Human Use (ICH), which brings together the regulatory authorities and pharmaceutical industry of Europe, Japan and the USA [44]. Legally-binding quality standards are also laid down in compendial monographs, the European Pharmacopoeia, the United States Pharmacopoeia and other country-specific compendia.

Once a vaccine has been approved for marketing, the manufacturer must adhere to quality assurance programs to evaluate various steps of the manufacturing process: from raw materials, through each stage of component preparation to the final formulated, filled and packaged product. Characterization and testing of raw materials are required, and the production procedure must be validated, documented and meet good manufacturing practice (GMP) requirements [52]. GMP covers the methods to be used in and the facilities or controls to be used for the manufacture, processing or packing of a drug to ensure its safety, quality and purity [44]. Final product testing covers aspects such as sterility, general safety, purity, identity and potency. The vaccine manufacturer must also demonstrate production consistency. For each lot of vaccine that is produced, release by national regulatory authorities provides a final check on the manufacturer’s performance in the control of the production process and may include additional, independent testing of sampled lots before approval for release and distribution by the manufacturer [53]. Inevitably, there are occasional disruptions of supply when these multiple steps of control of the production process do not permit a component to be used in a next step. Significant supply disruptions should be notified to authorities and can provide an indication of the reliability of the manufacturing processes.

A further oversight to industry processes is delivered through facility inspections by regulatory authorities. Manufacturers that fail to meet product standards or do not comply with GMP may be subject to product recalls, license suspension or withdrawal or even, in exceptional circumstances, closure of the production plant [54,55,56,57]. 

### 2.3. Vaccine Safety Surveillance: Pharmacovigilance

Safety is monitored throughout the vaccine’s lifecycle, starting at the time of preclinical evaluation and continued during clinical development and, following licensure, is monitored indefinitely while in use in immunization programs. The benefit-risk profile of the vaccine is therefore re-assessed constantly. Safety surveillance is the responsibility of not only those who develop or manufacture the products, but also those who are involved in vaccine distribution and administration [16,58]. For instance, the role of healthcare providers is essential in observing and reporting adverse events. Safety surveillance also requires close collaboration between regulators and industry [59].

Similar to the vaccine development and manufacturing processes, safety reporting requirements are guided by rules and regulations. For example, the EMA requires public release of vaccine safety information via Periodic Safety Update Reports (PSURs), which present the vaccine manufacturer’s integrated assessment of benefit-risk and exposure and are provided to the EMA for their assessment. From 2013, PSUR-related documents will be published on the EMA website, increasing the transparency of available safety information [60]. 

National and supranational vaccine pharmacovigilance programs involve, at the most basic level, passive surveillance of adverse events following immunization (AEFI), in which spontaneous AEFI reports are made to regulatory monitoring organizations, such as the Vaccine Adverse Event Reporting System (VAERS) in the USA and EudraVigilance in Europe [48,61,62]. Passive surveillance, therefore, relies on the detection and reporting of cases by healthcare providers. 

Where a safety signal is detected or when a new vaccine is introduced, passive surveillance of AEFI is not sufficient, and an “active” vaccine pharmacovigilance is established that involves prospective case finding and data collection [16]. Epidemiological studies may be required to assess whether an AEFI is causally related to a vaccine, as well as pathological or laboratory studies. In the USA, the CDC-sponsored Vaccine Safety Datalink (VSD) system enables active vaccine pharmacovigilance with a near real-time vaccine safety surveillance system via weekly data and sequential statistical analysis [63,64]. In Europe, the Vaccine Adverse Event Surveillance and Communication (VAESCO) project conducts similar vaccine safety studies to complement routine reporting of AEFI to EudraVigilance [65]. The Innovative Medicines Initiative is developing a public-private collaborative framework for rapid assessment of the benefit-risk profile of vaccines [66]. For countries that lack the infrastructure or resources necessary to carry out appropriate pharmacovigilance studies, the WHO Global Advisory Committee on Vaccine Safety (GACVS) provides independent, scientifically rigorous advice on vaccine safety issues of potential global importance [67]. Access to a global network for safety data exchange would also be beneficial [16,68]. National or supranational expert committees often advise local authorities on the nature of the observed events and, where a causal link is established, recommend actions to treat or manage the AEFI at a local level, where appropriate. The national or supranational regulatory authority also decides if the vaccine should be withdrawn from the market or if changes should be made to its licensed indications and safety warnings added to its prescribing information.

Active surveillance is important to evaluate the background incidence of rare conditions and autoimmune disorders to determine whether events that are temporally associated with vaccination are occurring at a higher rate than would be expected based upon the background incidence rate for that event [47]. This is essential to address public concerns and to provide accurate and reliable information on vaccines. For example, a tetravalent rhesus-human reassortant rotavirus vaccine licensed in the USA, *Rotashield*™* (Wyeth-Lederle Vaccines, Madison, NJ, USA; now owned by Pfizer, New York, NY, USA), was withdrawn from the market because of an association with intussusception that was identified through passive surveillance [69] and confirmed following collaboration of the ACIP, industry and managed care organizations [70]. This triggered active surveillance of the two currently available rotavirus vaccines, Rotarix™* (GlaxoSmithKline Vaccines, Wavre, Belgium) and *RotaTeq*^®^* (Merck and Co., Inc., Whitehouse Station, NJ, USA) and results have been published from large post-marketing studies conducted in Mexico, Brazil, Australia and the U.S. [71,72,73,74,75]. In its 2013 position statement, the WHO concluded from these data that in some, but not all, settings, a small increased risk of intussusception (about one to two per 100,000 infants vaccinated) was detected shortly after the first dose of both vaccines [76]. Where present, this risk is five to 10 times lower than that associated with the previous rotavirus vaccine that was withdrawn. This AEFI is mentioned as a precaution in the summary of product characteristics for both vaccines, and surveillance for intussusception continues. Current evidence, however, suggests that the benefits of the rotavirus vaccines, in terms of averted deaths and hospitalizations [76,77,78,79], outweigh the risk for intussusception. 

### 2.4. Disease Surveillance, Vaccine Impact and Uptake

Data on the efficacy of the vaccine in preventing disease in immunized populations are obtained from controlled studies. Vaccine effectiveness describes protection under programmatic implementation, reflecting the performance of the vaccine in the actual target population, and is generally monitored as part of post-marketing disease surveillance activities [49]. National and international disease surveillance, together with data on vaccination uptake, serve to document the impact of immunization programs. The effectiveness of current vaccines and vaccination policies, such as for a child immunization program, is mainly evaluated through continued passive surveillance, *i.e.*, by routinely reported data from an existing health system. These data may highlight the need for changes in program strategies, for example, from observations of disease trends over time or outbreak patterns from vaccine-preventable diseases. Passive surveillance data are normally evaluated and reported annually in national reports and at a supranational level [80].

An additional active surveillance approach, which is more specific and sensitive and also more resource-demanding, may be implemented and is the tool of choice to monitor and evaluate the impact on a vaccine-preventable disease that is targeted for eradication. An example is the active surveillance of acute flaccid paralysis in children aged under 15 years, as recommended by the WHO, to document progress towards reaching the target of polio eradication [81].

Information on vaccine uptake is crucial to evaluate the effectiveness of a vaccine or an immunization program. The best quality data are obtained on a real-time and case-based level, but as vaccines are distributed daily and in large numbers, it is highly resource-demanding to obtain data of this quality in all settings. Hence, vaccination coverage assessment may differ from one country to another in terms of the information system used, timeliness of reporting and data analysis methodology [82]. Therefore, to fully understand reports on vaccine effectiveness and impact, taking the heterogeneity of national systems and outputs into consideration, it is important to understand the origin of the data, such as type and timeliness of surveillance, data specifications and analysis methodology, especially when comparisons are made among national reports. 

### 2.5. Community Engagement

To achieve high uptake, there must be broad acceptance of the medical need and safety of immunization, as well as the availability of acceptable health systems that support vaccine delivery. However, the process of developing benefit-risk communication messages to instill public trust is complicated, requiring different types of research at a local community level for each new vaccine introduced. In particular, advocacy and communication strategies must be tailored to the population concerned, as has been demonstrated with the introduction of vaccines against human papillomavirus (HPV). Before licensing, information-gathering exercises showed the importance of raising awareness of HPV as a cause of cervical cancer before introducing an immunization program, the success of which was dependent on targeting primarily young adolescent girls before HPV exposure [83,84]. For example, interviews held with parents of children aged eight to 10 years in the UK before the introduction of the HPV vaccination program showed that most had not heard of HPV and were not aware of its role in cervical cancer [85]. There were also concerns about offering a vaccine that protects against a sexually transmitted infection to children and that the vaccine should be offered at an older age in conjunction with a sex education program. This perceived lack of need and misunderstanding about the optimal time for vaccination, along with some safety concerns, have contributed to low vaccine uptake in some countries [86,87,88]. 

For vaccine programs to be successful, it is important to present facts about disease burden and vaccine prevention in an accurate, appropriate and easy to understand way, including clear explanation of the risks of disease *versus* the risks of vaccination (Table 2). Healthcare providers have a central role in maintaining public trust in vaccination through direct communication with the vaccine or the vaccinated child’s parent [13,89,90]. Gaps in knowledge and poor communication from healthcare providers are detrimental to high immunization rates [90], and it is important that healthcare providers have confidence in the information they provide. Stakeholders need to be engaged in a manner that takes into account the knowledge, attitudes, behaviors and values of the local population [12,91]. 

**Table 2:** An example vaccine information statement (VIS) for the measles, mumps and rubella (MMR) vaccine (adapted from VIS produced by the CDC [92] and data in the CDC “Pink Book” [93]).

MMR disease and complications	MMR vaccine and complications
**Measles** Measles virus causes rash, cough, runny nose, eye irritation and fever. Complications include: Ear infection (7 persons out of 100) * Pneumonia (6 persons out of 100) * Seizures (jerking and staring) (6 to 7 persons out of 1,000) * Death (2 persons out of 1,000) *	Children should receive 2 doses of MMR vaccine. Complications include: *Mild problems* Fever (up to 1 person out of 6) Mild rash (about 1 person out of 20) Swelling of glands in the cheeks or neck (about 1 person out of 75) *Moderate problems* Seizure (jerking or staring) caused by fever (about 1 out of 3,000 doses) Temporary pain and stiffness in the joints, mostly in teenage or adult women (up to 1 out of 4) Temporary low platelet count, which can cause a bleeding disorder (about 1 out of 30,000 doses) *Severe problems* Serious allergic reaction (less than 1 out of a million doses) Several other severe problems have been reported after a child gets MMR vaccine, including deafness, long-term seizures, coma or lowered consciousness, permanent brain damage These are so rare that it is hard to tell whether they are caused by the vaccine
**Mumps** Mumps virus causes fever, headache, muscle pain, loss of appetite and swollen glands. Complications include: Deafness (1 person out of 20,000) * Meningitis (infection of the brain and spinal cord covering) (15 persons out of 100) * Painful swelling of the testicles or ovaries (adults: 1 person out of 2 or 3); rarely sterility	
**Rubella** Rubella virus causes rash, arthritis (mostly in women) and mild fever. Complications include: Encephalitis (1 person out of 6,000) ** Hemorrhagic manifestations (1 person out of 3,000) ** If a woman gets rubella while she is pregnant, she could have a miscarriage or her baby could be born with serious birth defects	

* Rates reported for measles and mumps complications are from CDC Pink Book; ** complications for rubella are not presented in the VIS and have been sourced from the CDC Pink Book.

Therefore, the confidence of healthcare providers in vaccines should be reinforced by better and deeper scientific and public health training on vaccines during their medical studies and through postgraduate education. The WHO has identified several areas that need to be addressed to ensure healthcare providers are well prepared for immunization sessions [94]. These include strengthening of pre-service training within the faculties of medicine, nursing, pharmacy and public health, as well as in-service training through specific support and regular refresher courses, such as vaccinology courses. Moreover, delivery of effective benefit-risk communication messages is highly dependent on a strong immunization program in which policies are driven by processes for developing, manufacturing and monitoring the safety and effectiveness of vaccines that are transparent, *i.e.*, are performed with openness, so that people can trust that they are fair and honest.

### 2.6. Crisis Management

Although vaccine safety crises are uncommon, they have the potential to disrupt immunization activities and, thereby, affect public health. It is, therefore, important to ensure that vaccine safety communication plans are in place at an early stage, as recommended in the Global Vaccine Safety Initiative developed by the WHO and a group of partners [16,58]. This should include a mechanism for providing feedback to vaccines or parents and healthcare providers on specific concerns raised about vaccines, such as a reported AEFI; not having such feedback could reinforce the subconscious sense that vaccines are dangerous [24].

A number of false vaccine concerns have been reported that have fuelled the effectiveness of anti-vaccine advocates. One of the most notable vaccination controversies was related to the presumed link between measles, mumps and rubella (MMR) vaccination and autism in children, which was initiated by an article in *The Lancet* by Wakefield *et al.* in 1998 (article retracted) proposing an association between MMR and autism [95]. Despite numerous studies that failed to show a link between the MMR vaccine and autism, media coverage of the allegations was vast, and a significant decrease in vaccination trust and vaccine coverage occurred and still remains in various countries [31,32]. In other cases, immunization programs have been suspended in response to false vaccine concerns, such as the reported link between multiple sclerosis and hepatitis B vaccination that led to the French government suspending, temporarily, the school-based hepatitis B immunization program in 1998 [96]; and disrupted immunization in various countries due to a loss of confidence in pertussis vaccination in the 1970s and 1980s [97]. In both cases, vaccine hesitancy was proven to be unfounded [96,97,98]. 

Vaccine beliefs should be based on accurate, factual information regarding both the benefits and risks associated with vaccines [24], delivered with tailored advocacy and communication to explain medical need and improve knowledge of vaccine safety systems. This may require social mobilization, where people with influence, such as healthcare providers, local authorities, religious leaders, teachers and community leaders, are involved in addressing local social and behavioral aspects of vaccine hesitancy [99,100]. In the future, the Internet and social media are likely to be used more frequently during the launch of immunization campaigns to prevent misinformation [29,30,31]. This might include web-based decision aids, which have been shown to improve attitudes to vaccination [101]. It has been suggested that the interactive potential of the Internet could be harnessed even further by integrating factual information with people’s own values and preferences, allowing users to receive personalized vaccination recommendations [23]. Strategies for identifying early warning signals of areas of decreased confidence would also encourage concerns to be addressed efficiently, such as the Vaccine Confidence Index developed by the London School of Hygiene and Tropical Medicine [102].

### 2.7. Public Disclosure and Transparency

According to a survey of over 1,200 parents in the USA, vaccine manufacturers were considered a good or excellent source of information by only 29% of those who did not use the Internet as a source of vaccine information and by 23% of those who did use the Internet [26]. Healthcare providers were regarded as a good source of information by 85% and 69% of those surveyed, respectively. This lack of trust in vaccine manufacturers is possibly linked to the fact that the vaccine industry profits from selling vaccines and that the scientists who develop and test vaccines, as well as the doctors who promote them, are perceived to have a vested interest in highlighting their benefits [13]. Consequently, the public may feel that vaccine manufacturer employees, including research scientists, have a conflict of interest in providing the “full picture” in regards to their vaccines’ benefit-risk profiles and knowledge gaps [103]. Moreover, concerns raised in the past, regarding issues, such as bias in the publication of trial results [104,105,106,107,108], the relationship between industry and the medical profession and patients’ organizations [109,110,111,112] and the regulation of promotional activities and materials [113], have had a negative impact on information provided by the pharmaceutical industry. 

Openness and honesty attained with bi-directional communication with healthcare providers and the general population is one of the factors that determines trust in industry [114]. It therefore follows that manufacturers of vaccines should provide product information that is accessible to all those interested in vaccine implementation, including the general population. Vaccine manufacturers now post information on the protocols and results of vaccine clinical trials online to meet International Committee of Medical Journal Editors’ requirements [115]. ClinicalTrials.gov, run by the USA National Institutes of Health [116], was the first online registry for clinical trials and is the largest and most widely used. 

There have been calls to go further and for relevant anonymized patient-level data to be made available upon request for all clinical trials of drugs and devices; since January 2013, the British Medical Journal requires this commitment before publication can be considered [117]. Several pharmaceutical companies have also committed to seek publication of results from all sponsored clinical trials that evaluate their medicines in peer-reviewed scientific journals. In October 2012, GlaxoSmithKline announced that it will make detailed anonymized patient-level data from their clinical trials available to qualified researchers through SHARE (Sharing Anonymized Research Data) [118,119]. Disclosure of comprehensive clinical study results by the vaccine industry would also be desirable [120].

Openness and transparency in the exchange of information between the vaccine industry and other stakeholders in vaccine implementation should help to re-build trust and, hence, maintain confidence in immunization programs. Also, increased access to data by outside experts will allow independent trial analysis, offering the potential for different perspectives on the results, which could lead to improvements in trial design or aspects of the immunization program or even the development of novel medicines [103,118].

## 3. Conclusions: The Need for Partnerships

In this Decade of Vaccines, the expansion of immunization programs is necessary to meet the goals of healthcare organizations worldwide and to improve health at a country level. In a world of networks, the vaccination debate has moved from the classical top-down model of information provision to one of social media debate, where each contributor is seen as an equal player. However, websites often present suboptimal and inaccurate information, and as a result, public concerns surrounding vaccinations (most frequently, vaccine safety) have the potential to spread rapidly. Misconceptions and misunderstandings surrounding vaccines have been fuelled by the difficulty of many stakeholders in communicating and informing others in effective ways, be it parents, vaccine recipients or healthcare providers. 

No medical intervention, including vaccines, is completely safe and without adverse events. The most common AEFIs are time-limited and mild and continuous benefit-risk evaluations are performed by regulatory authorities, using the vaccine safety evaluation and monitoring systems that are in place to identify safety concerns. Serious adverse events and associated risks are assessed and compared to the benefits of vaccination. Even so, reassurances on vaccine safety and of the necessity for vaccination via evidence-based recommendations and policies, as well as stringent safety and disease surveillance procedures, are not sufficient to counter all negative beliefs surrounding vaccines. Further social and behavior science research is needed to determine how to address beliefs appropriately via effective benefit-risk communication messages. Reactions to issues related to vaccine production, safety or implementation programs must be handled with speed and must also be fair, balanced and accurate in the assessment and in all communications. In particular, unexpected and/or serious AEFIs require rapid and comprehensive investigation to provide effective and transparent information regarding causality and management. This necessitates ongoing education and a commitment to communication and dialogue among all involved stakeholders. 

Importantly, in view of their central role in maintaining public trust in vaccines, healthcare providers need to feel confident in providing advice and need to be updated in a timely manner when scares arise. This might require an increased amount of time spent on the topic of immunization in medical and nursing schools and an increased focus on continued medical education in this area. Better tailored communication materials shared through appropriate channels would further support community engagement. 

Health authorities need to play an important role in implementing vaccine communication plans and in the wider field of transparency and accountability in vaccine decision-making. Confidence in industry requires a commitment to bring the same high quality and efficacious vaccines to all countries and a major role in the transparent infrastructure for developing, manufacturing and monitoring the safety and effectiveness of vaccines. The vaccines industry must ensure that accurate and reliable information is provided on its products in order to address vaccine hesitancy associated with social and behavioral issues. The industry should also have strategies for providing accurate and reliable information on the benefit-risk profile of vaccines, which are appropriate for each audience concerned and supported by evidence-based and transparent recommendations. This would help other stakeholders to view the pharmaceutical industry as an integral partner in public health issues. Moreover, interactions among vaccination stakeholders should be guided by the highest standards and codes of conduct in order to address any concerns surrounding the impartiality of vaccine manufacturers in terms of benefit-risk communication. 

This review provides a broad overview of the systems that contribute to building confidence in vaccines and is therefore limited in terms of detail on specific topics. It is clear, however, that to sustain trust in vaccines, partnerships between all stakeholders in the public health sector, such as health authorities, policy makers, national and supranational organizations, healthcare providers, vaccine manufacturers and others, are needed to ensure high vaccine uptake rates, identify and allow the introduction of new vaccines and inform the public and others of the benefits of vaccines and how vaccine safety is constantly assessed, assured and communicated. The development of effective benefit-risk communication messages to instill public trust in vaccines is complex, but can be achieved with collaborative and transparent approaches, thereby encouraging the success of immunization programs.

## Trademark Statement

Rotarix is a trademark of the GlaxoSmithKline group of companies. RotaShield is a trademark of Wyeth-Lederle Vaccines, and RotaTeq is a trademark of Merck and Co., Inc.

## References

[1] Duclos P., Okwo-Bele J.M., Gacic-Dobo M., Cherian T. (2009). Global immunization: Status, progress, challenges and future. BMC Int. Health Hum. Rights.

[2] World Health Organization (2011). Global routine vaccination coverage, 2010. Wkly. Epidemiol. Rec..

[3] Roush S.W., Murphy T.V. (2007). Historical comparisons of morbidity and mortality for vaccine-preventable diseases in the United States. JAMA.

[4] WHO, UNICEF, World Bank (2009). State of the World’s Vaccines and Immunization.

[5] Ozawa S., Mirelman A., Stack M.L., Walker D.G., Levine O.S. (2012). Cost-effectiveness and economic benefits of vaccines in low- and middle-income countries: A systematic review. Vaccine.

[6] Stack M.L., Ozawa S., Bishai D.M., Mirelman A., Tam Y., Niessen L., Walker D.G., Levine O.S. (2011). Estimated economic benefits during the “decade of vaccines” include treatment savings, gains in labor productivity. Health Aff. (Millwood).

[7] Bonanni P., Santos J., Garçon N., Stern P.L., Cunningham A.L., Stanberry L.R. (2011). Vaccine Evolution. Understanding Modern Vaccines: Perspectives in Vaccinology.

[8] Blank P.R., Schwenkglenks M., Szucs T.D. (2009). Disparities in influenza vaccination coverage rates by target group in five European countries: Trends over seven consecutive seasons. Infection.

[9] Centers for Disease Control and Prevention (2011). Global routine vaccination coverage, 2010. Morb. Mortal. Wkly. Rep..

[10] World Health Organization (2012). Draft Global Vaccine Action Plan. Report by the Secretariat.

[11] Moxon E.R., Das P., Greenwood B., Heymann D.L., Horton R., Levine O.S., Plotkin S., Nossal G. (2011). A call to action for the new decade of vaccines. Lancet.

[12] Bill & Melinda Gates Foundation Bill and Melinda Gates pledge $10 billion in call for Decade of Vaccines. Increased vaccination could save more than 8 million children by 2020; significant funding gaps remain, others must join effort. http://www.gatesfoundation.org/press-releases/Pages/decade-of-vaccines-wec-announcement-100129.aspx.

[13] Larson H.J., Cooper L.Z., Eskola J., Katz S.L., Ratzan S. (2011). Addressing the vaccine confidence gap. Lancet.

[14] Burnett R.J., Larson H.J., Moloi M.H., Tshatsinde E.A., Meheus A., Paterson P., Francois G. (2012). Addressing public questioning and concerns about vaccination in South Africa: A guide for healthcare workers. Vaccine.

[15] Glanz J.M., Newcomer S.R., Narwaney K.J., Hambidge S.J., Daley M.F., Wagner N.M., McClure D.L., Xu S., Rowhani-Rahbar A., Lee G.M. (2013). A population-based cohort study of undervaccination in 8 managed care organizations across the United States. JAMA.

[16] Kim T.H., Johnstone J., Loeb M. (2011). Vaccine herd effect. Scand. J. Infect. Dis..

[17] World Health Organization (2012). Global Vaccine Safety Blueprint.

[18] Pan American Health Organization (2002). Immunization Safety. How to Address Events Allegedly Attributable to Vaccination or Immunization.

[19] Lantos J.D., Jackson M.A., Opel D.J., Marcuse E.K., Myers A.L., Connelly B.L. (2010). Controversies in vaccine mandates. Curr. Probl. Pediatr. Adolesc. Health Care.

[20] Stanton B.F. (2004). Assessment of relevant cultural considerations is essential for the success of a vaccine. J. Health Popul. Nutr..

[21] Lau C.Y., Stansbury J.P., Gust D.A., Kafaar Z. (2009). Social and behavioral science in HIV vaccine trials: A gap assessment of the literature. Expert Rev. Vaccines.

[22] Funk S., Salathé M., Jansen V.A. (2010). Modelling the influence of human behaviour on the spread of infectious diseases: A review. J. R. Soc. Interface.

[23] Myers L.B., Goodwin R. (2011). Determinants of adults’ intention to vaccinate against pandemic swine flu. BMC Public Health.

[24] Connolly T., Reb J. (2012). Toward interactive, Internet-based decision aid for vaccination decisions: Better information alone is not enough. Vaccine.

[25] MacDonald N.E., Smith J., Appleton M. (2012). Risk perception, risk management and safety assessment: What can governments do to increase public confidence in their vaccine system?. Biologicals.

[26] Muscat M. (2011). Who gets measles in Europe?. J. Infect. Dis..

[27] Jones A.M., Omer S.B., Bednarczyk R.A., Halsey N.A., Moulton L.H., Salmon D.A. (2012). Parents’ source of vaccine information and impact on vaccine attitudes, beliefs, and nonmedical exemptions. Adv. Prev. Med..

[28] Centers for Disease Control and Prevention Some common misconceptions about vaccination and how to respond to them. http://www.cdc.gov/print.do?url=http://www.cdc.gov/vaccines/vac-gen/6mishome.htm/.

[29] Betsch C., Brewer N.T., Brocard P., Davies P., Gaissmaier W., Haase N., Leask J., Renkewitz F., Renner B., Reyna V.F. (2012). Opportunities and challenges of Web 2.0 for vaccination decisions. Vaccine.

[30] Betsch C., Sachse K. (2012). Dr. Jekyll or Mr. Hyde? (How) the Internet influences vaccination decisions: Recent evidence and tentative guidelines for online vaccine communication. Vaccine.

[31] Kata A. (2012). Anti-vaccine activists, Web 2.0, and the postmodern paradigm—An overview of tactics and tropes used online by the anti-vaccination movement. Vaccine.

[32] Poland G.A., Jacobson R.M. (2012). The clinician’s guide to the anti-vaccinationists’ galaxy. Hum. Immunol..

[33] Carrillo-Santisteve P., Lopalco P.L. (2012). Measles still spreads in Europe: Who is responsible for the failure to vaccinate?. Clin. Microbiol. Infect..

[34] Muscat M., Bang H., Glismann S. (2008). Measles is still a cause for concern in Europe. Eur. Surveill..

[35] Jegede A.S. (2007). What led to the Nigerian boycott of the polio vaccination campaign?. PLoS Med..

[36] Centers for Disease Control and Prevention (2012). Progress toward poliomyelitis eradication—Nigeria, January 2011–September 2012. MMWR Morb. Mortal. Wkly. Rep..

[37] Taylor K., Nguyen A., Stéphenne J. (2009). The need for new vaccines. Vaccine.

[38] Kennedy A., Lavail K., Nowak G., Basket M., Landry S. (2011). Confidence about vaccines in the United States: Understanding parents’ perceptions. Health Aff. (Millwood).

[39] Blecher M.S., Meheus F., Kollipara A., Hecht R., Cameron N.A., Pillay Y., Hanna L. (2012). Financing vaccinations—The South African experience. Vaccine.

[40] Shen A.K., Spinner J.R., Salmon D.A., Gellin B.G. (2011). Strengthening the U.S. vaccine and immunization enterprise: The role of the National Vaccine Advisory Committee. Public Health Rep..

[41] Duclos P., Ortynsky S., Abeysinghe N., Cakmak N., Janusz C.B., Jauregui B., Mihigo R., Mosina L., Sadr-Azodi N., Takashima Y. (2012). Monitoring of progress in the establishment and strengthening of national immunization technical advisory groups. Vaccine.

[42] Bridges C.B., Woods L., Coyne-Beasley T. (2013). Advisory Committee on Immunization Practices (ACIP) recommended immunization schedule for adults aged 19 years and older—United States, 2013. Morb. Mortal. Wkly. Rep..

[43] Akinsanya-Beysolow I., Jenkins R., Meissner H.C. (2013). Advisory Committee on Immunization Practices (ACIP) recommended immunization schedule for persons aged 0 through 18 years—United States, 2013. Morb. Mortal. Wkly. Rep..

[44] Marshall V., Baylor N.W. (2011). Food and Drug Administration regulation and evaluation of vaccines. Pediatrics.

[45] Lebron J.A., Wolf J.J., Kaplanski C.V., Ledwith B.J. (2005). Ensuring the quality, potency and safety of vaccines during preclinical development. Expert Rev. Vaccines.

[46] European Medicines Agency (2009). Guideline on the Conduct of Pharmacovigilance for Vaccines for Pre- and Post-exposure Prophylaxis against Infectious Diseases.

[47] Leroux-Roels G., Bonanni P., Tantawichien T., Zepp F., Garçon N., Stern P.L., Cunningham A.L., Stanberry L.R. (2011). Vaccine Development. Understanding Modern Vaccines: Perspectives in Vaccinology.

[48] Bonhoeffer J., Black S., Izurieta H., Zuber P., Sturkenboom M. (2012). Current status and future directions of post-marketing vaccine safety monitoring with focus on USA and Europe. Biologicals.

[49] World Health Organization (2005). Vaccine Introduction Guidelines. Adding a Vaccine to a National Immunization Program: Decision and Implementation.

[50] The National Vaccine Advisory Committee A report of the National Vaccine Advisory Committee. Strengthening the supply of routinely recommended vaccines in the United States. http://www.hhs.gov/nvpo/nvac/nvac-vsr.html#problems/.

[51] Strugnell R., Zepp F., Cunningham A.L., Tantawichien T., Garçon N., Stern P.L., Cunningham A.L., Stanberry L.R. (2011). Vaccine Antigens. Understanding Modern Vaccines: Perspectives in Vaccinology.

[52] Minor P. (2012). Considerations for setting the specifications of vaccines. Expert Rev. Vaccines.

[53] Dellepiane N., Griffiths E., Milstien J.B. (2000). New challenges in assuring vaccine quality. Bull. World Health Organ..

[54] Wilkie D. The Chiron case: Good Manufacturing Practice gone bad. http://www.the-scientist.com/?articles.view/articleNo/16290/title/The-Chiron-Case--Good-Manufacturing-Practice-Gone-Bad/.

[55] MacDonald G. GSK pulls vaccine batch in Canada over contamination concerns. http://www.in-pharmatechnologist.com/Regulatory-Safety/GSK-pulls-vaccine-batch-in-Canada-over-contamination-concerns/.

[56] Taylor N. India plans $37m investment in vaccine cGMP compliance. http://www.in-pharmatechnologist.com/Processing/India-plans-37m-investment-in-vaccine-cGMP-compliance/.

[57] Centers for Disease Control and Prevention Recalled vaccines. http://www.cdc.gov/vaccines/recs/recalls/default.htm/.

[58] World Health Organization The Global Vaccine Safety Initiative (GVSI). http://www.who.int/vaccine_safety/initiative/en/.

[59] ICH (2004). ICH Harmonised Tripartite Guideline.

[60] European Medicines Agency (2012). Guideline on Good Pharmacovigilance Practices (GVP). Module VII—Periodic Safety Update Report.

[61] Chen R.T., Rastogi S.C., Mullen J.R., Hayes S.W., Cochi S.L., Donlon J.A., Wassilak S.G. (1994). The Vaccine Adverse Event Reporting System (VAERS). Vaccine.

[62] European Medicines Agency EudraVigilance. http://eudravigilance.ema.europa.eu/human/index.asp.

[63] Lieu T.A., Kulldorff M., Davis R.L., Lewis E.M., Weintraub E., Yih K., Yin R., Brown J.S., Platt R. (2007). Real-time vaccine safety surveillance for the early detection of adverse events. Med. Care.

[64] Baggs J., Gee J., Lewis E., Fowler G., Benson P., Lieu T., Naleway A., Klein N.P., Baxter R., Belongia E. (2011). The Vaccine Safety Datalink: A model for monitoring immunization safety. Pediatrics.

[65] Eurosurveillance Editorial Team (2009). ECDC in collaboration with the VAESCO consortium to develop a complementary tool for vaccine safety monitoring in Europe. Euro Surveill..

[66] Laverty H., Gunn M., Goldman M. (2012). Improving R&D productivity of pharmaceutical companies through public-private partnership: Experiences from the Innovative Medicines Initiative. Expert Rev. Pharmacoecon. Outcomes Res..

[67] World Health Organization (2010). Global Advisory Committee on Vaccine Safety, 3–4 December 2009. Wkly. Epidemiol. Rec..

[68] Graham J.E., Borda-Rodriguez A., Huzair F., Zinck E. (2012). Capacity for a global vaccine safety system: The perspective of national regulatory authorities. Vaccine.

[69] Centers for Disease Control and Prevention (1999). Intussusception among recipients of rotavirus vaccine—United States, 1998–1999. Morb. Mortal. Wkly. Rep..

[70] Centers for Disease Control and Prevention (1999). Withdrawal of rotavirus vaccine recommendation. Morb. Mortal. Wkly. Rep..

[71] Velázquez F.R., Colindres R.E., Grajales C., Hernández M.T., Mercadillo M.G., Torres F.J., Cervantes-Apolinar M., DeAntonio-Suarez R., Ortega-Barria E., Blum M. (2012). Postmarketing surveillance of intussusception following mass introduction of the attenuated human rotavirus vaccine in Mexico. Pediatr. Infect. Dis. J..

[72] Shui I.M., Baggs J., Patel M., Parashar U.D., Rett M., Belongia E.A., Hambidge S.J., Glanz J.M., Klein N.P., Weintraub E. (2012). Risk of intussusception following administration of a pentavalent rotavirus vaccine in U.S. infants. JAMA.

[73] Buttery J.P., Danchin M.H., Lee K.J., Carlin J.B., McIntyre P.B., Elliott E.J., Booy R., Bines J.E. (2011). Intussusception following rotavirus vaccine administration: Post-marketing surveillance in the National Immunization Program in Australia. Vaccine.

[74] Patel M.M., López-Collada V.R., Bulhões M.M., de Oliveira L.H., Bautista Márquez A., Flannery B., Esparza-Aguilar M., Montenegro Renoiner E.I., Luna-Cruz M.E., Sato H.K. (2011). Intussusception risk and health benefits of rotavirus vaccination in Mexico and Brazil. N. Engl. J. Med..

[75] Loughlin J., Mast T.C., Doherty M.C., Wang F.T., Wong J., Seeger J.D. (2012). Postmarketing evaluation of the short-term safety of the pentavalent rotavirus vaccine. Pediatr. Infect. Dis. J..

[76] World Health Organization (2013). Rotavirus vaccines. WHO position paper—January 2013. Wkly. Epidemiol. Rec..

[77] Centers for Disease Control and Prevention (2011). Progress in the introduction of rotavirus vaccine—Latin America and the Caribbean, 2006–2010. Morb. Mortal. Wkly. Rep..

[78] Tate J.E., Patel M.M., Cortese M.M., Lopman B.A., Gentsch J.R., Fleming J., Steele A.D., Parashar U.D. (2012). Remaining issues and challenges for rotavirus vaccine in preventing global childhood diarrheal morbidity and mortality. Expert Rev. Vaccines.

[79] Gastañaduy P.A., Sánchez-Uribe E., Esparza-Aguilar M., Desai R., Parashar U.D., Patel M., Richardson V. (2013). Effect of rotavirus vaccine on diarrhea mortality in different socioeconomic regions of Mexico. Pediatrics.

[80] World Health Organization Immunization surveillance, assessment and monitoring. WHO/UNICEF joint reporting process. http://www.who.int/immunization_monitoring/routine/joint_reporting/en/index.html#/.

[81] World Health Organization (2003). WHO-recommended Standards for Surveillance of Selected Vaccine-preventable Diseases.

[82] VENICE II Consortium Vaccination coverage assessment in EU/EEA, 2011. http://venice.cineca.org/Final_Vaccination_Coverage_Assesment_Survey_2011_1.pdf.

[83] Bonanni P., Levi M., Latham N.B., Bechini A., Tiscione E., Lai P., Panatto D., Gasparini R., Boccalini S. (2011). An overview on the implementation of HPV vaccination in Europe. Hum. Vaccin..

[84] World Health Organization (2009). Human papillomavirus vaccines. WHO position paper. Wkly. Epidemiol. Rec..

[85] Noakes K., Yarwood J., Salisbury D. (2006). Parental response to the introduction of a vaccine against human papilloma virus. Hum. Vaccin..

[86] Markowitz L.E., Tsu V., Deeks S.L., Cubie H., Wang S.A., Vicari A.S., Brotherton J.M. (2012). Human papillomavirus vaccine introduction—The first five years. Vaccine.

[87] Kane M.A., Serrano B., de Sanjose S., Wittet S. (2012). Implementation of human papillomavirus immunization in the developing world. Vaccine.

[88] Watson-Jones D., Tomlin K., Remes P., Baisley K., Ponsiano R., Soteli S., de Sanjose S., Changalucha J., Kapiga S., Hayes R.J. (2012). Reasons for receiving or not receiving HPV vaccination in primary schoolgirls in Tanzania: A case control study. PLoS One.

[89] Leask J., Kinnersley P., Jackson C., Cheater F., Bedford H., Rowles G. (2012). Communicating with parents about vaccination: A framework for health professionals. BMC Pediatr..

[90] Simone B., Carrillo-Santisteve P., Lopalco P.L. (2012). Healthcare workers role in keeping MMR vaccination uptake high in Europe: A review of evidence. Euro Surveill..

[91] Steben M., Jeronimo J., Wittet S., Lamontagne D.S., Ogilvie G., Jensen C., Smith J., Franceschi S. (2012). Upgrading public health programs for human papillomavirus prevention and control is possible in low- and middle-income countries. Vaccine.

[92] Centers for Disease Control and Prevention MMR Vaccine. What You Need to Know. http://www.cdc.gov/vaccines/pubs/vis/downloads/vis-mmr.pdf.

[93] Centers for Disease Control and Prevention (2012). Measles; Mumps; Rubella. Epidemiology and Prevention of Vaccine-preventable Diseases.

[94] World Health Organization 3rd Global meeting on implementing new and under-utilized vaccines, 16–18 June 2009. Workgroup 8. Training of health staff and review of the Global Immunization Training Framework. http://www.who.int/nuvi/2009_meeting_summary_training/en/.

[95] Wakefield A.J., Murch S.H., Anthony A., Linnell J., Casson D.M., Malik M., Berelowitz M., Dhillon A.P., Thomson M.A., Harvey P. (1998). Ileal-lymphoid-nodular hyperplasia, non-specific colitis, and pervasive developmental disorder in children. Lancet.

[96] Hall A., Kane M., Roure C., Meheus A. (1999). Multiple sclerosis and hepatitis B vaccine?. Vaccine.

[97] Gangarosa E.J., Galazka A.M., Wolfe C.R., Phillips L.M., Gangarosa R.E., Miller E., Chen R.T. (1998). Impact of anti-vaccine movements on pertussis control: The untold story. Lancet.

[98] Ascherio A., Zhang S., Hernan M., Olek M., Coplan P., Brodovicz K. (2001). Hepatitis B vaccination and the risk of multiple sclerosis: Case-control studies. Gastroenterol. Clin. Biol..

[99] Baleta A.F., van den Heever J., Burnett R.J. (2012). Meeting the need for advocacy, social mobilisation and communication in the introduction of three new vaccines in South Africa—Successes and challenges. Vaccine.

[100] World Health Organization (2012). Communication for Behavioural Impact (COMBI). A Toolkit for Behavioural and Social Communication in Outbreak Response.

[101] Wallace C., Leask J., Trevena L.J. (2006). Effects of a web based decision aid on parental attitudes to MMR vaccination: A before and after study. BMJ.

[102] London School of Hygiene & Tropical Medicine The Vaccine Confidence Project. About the VCI. http://www.vaccineconfidence.org/VCI.html/.

[103] Osterhaus A.D., Vanlangendonck C. (2012). About courageous scientists, responsible policy makers, bridge-builders and preparedness for the next influenza pandemic. Vaccine.

[104] Lexchin J., Bero L.A., Djulbegovic B., Clark O. (2003). Pharmaceutical industry sponsorship and research outcome and quality: Systematic review. BMJ.

[105] Smith R. (2003). Medical journals and pharmaceutical companies: Uneasy bedfellows. BMJ.

[106] Melander H., Ahlqvist-Rastad J., Meijer G., Beermann B. (2003). Evidence b(i)ased medicine—Selective reporting from studies sponsored by pharmaceutical industry: Review of studies in new drug applications. BMJ.

[107] Godlee F. (2009). Doctors, patients, and the drug industry. BMJ.

[108] Lundh A., Sismondo S., Lexchin J., Busuioc O.A., Bero L. (2012). Industry sponsorship and research outcome. Cochrane Database Syst. Rev..

[109] Herxheimer A. (2003). Relationships between the pharmaceutical industry and patients’ organisations. BMJ.

[110] Abbasi K., Smith R. (2003). No more free lunches. BMJ.

[111] Moynihan R. (2008). Is the relationship between pharma and medical education on the rocks?. BMJ.

[112] Moynihan R. (2008). Key opinion leaders: Independent experts or drug representatives in disguise?. BMJ.

[113] Coombes R. (2005). Drug industry’s new code criticised for lacking teeth. BMJ.

[114] Maeda Y., Miyahara M. (2003). Determinants of trust in industry, government, and citizen’s groups in Japan. Risk Anal..

[115] DeAngelis C.D., Drazen J.M., Frizelle F.A., Haug C., Hoey J., Horton R., Kotzin S., Laine C., Marusic A., Overbeke A.J. (2004). Clinical trial registration: A statement from the International Committee of Medical Journal Editors. JAMA.

[116] ClinicalTrials.gov, a service of the U.S. National Institutes of Health http://www.clinicaltrials.gov/.

[117] Godlee F., Groves T. (2012). The new BMJ policy on sharing data from drug and device trials. BMJ.

[118] (2012). Toward clinical transparency. Nat. Med..

[119] Godlee F. (2012). Clinical trial data for all drugs in current use. BMJ.

[120] Coombes R. (2013). Andrew Witty: The acceptable face of big pharma?. BMJ.

